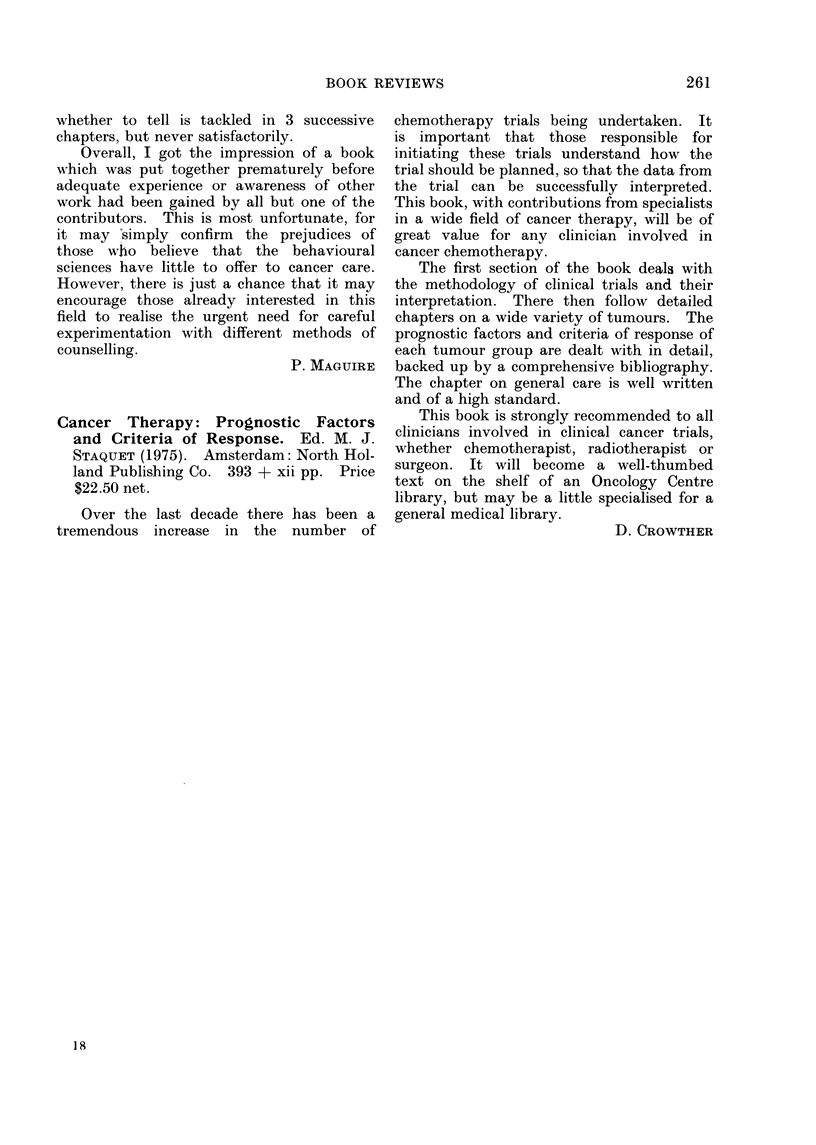# Cancer Therapy: Prognostic Factors and Criteria of Response

**Published:** 1977-02

**Authors:** D. Crowther


**Cancer Therapy: Prognostic Factors and Criteria of Response.** Ed. M. J. Staquet (1975). Amsterdam: North Holland Publishing Co. 393 + xii pp. Price $22.50 net.

Over the last decade there has been a tremendous increase in the number of chemotherapy trials being undertaken. It is important that those responsible for initiating these trials understand how the trial should be planned, so that the data from the trial can be successfully interpreted. This book, with contributions from specialists in a wide field of cancer therapy, will be of great value for any clinician involved in cancer chemotherapy.

The first section of the book deals with the methodology of clinical trials and their interpretation. There then follow detailed chapters on a wide variety of tumours. The prognostic factors and criteria of response of each tumour group are dealt with in detail, backed up by a comprehensive bibliography. The chapter on general care is well written and of a high standard.

This book is strongly recommended to all clinicians involved in clinical cancer trials, whether chemotherapist, radiotherapist or surgeon. It will become a well-thumbed text on the shelf of an Oncology Centre library, but may be a little specialised for a general medical library.

D. Crowther